# Blue Light Inhibits *E. coli*, but Decisive Parameters Remain Hidden in the Dark: Systematic Review and Meta-Analysis

**DOI:** 10.3389/fmicb.2022.867865

**Published:** 2022-04-08

**Authors:** Connor Lawrence, Sebastian Waechter, Beatrix W. Alsanius

**Affiliations:** ^1^Microbial Horticulture Unit, Department of Biosystems and Technology, Swedish University of Agricultural Sciences, Alnarp, Sweden; ^2^Department of Clinical Sciences, Lund University, Lund, Sweden

**Keywords:** death, exposure, inactivation, intensity, short wave blue light, strain, wavelength

## Abstract

Blue light (400–500 nm) alleviates overexposure risks associated to UV light and has therefore gained increased interest in multiple applications. This meta-analysis deals with decontamination of *E. coli* through the use of blue light based from nine recent publications identified *via* a systematic literature search. In these studies, various pathogenic and non-pathogenic *E. coli* strains grown in nutritional broths were exposed to wavelengths ranging from 395 to 460 nm. Five meta-analyses were performed using Cochrane’s software for meta-analyses (Review Manager): one including all studies to estimate the effect of *E. coli* reduction and four subgroup-analyses considering reported intensities, wavelengths, exposure dose as well as serovars/pathovars. Random effects models were used. All included studies used colony-forming units to estimate the impact of *E. coli* reduction. None of the included studies involved an organic matrix (e.g., skin, food related surface). Exposure to blue light had a significant and large reducing effect on viable counts of *E. coli*. However, substantial heterogeneity across studies was observed. Among subgroups, reported intensity and wavelength showed the clearest impact on *E. coli* reduction. With respect to the reported exposure dose, the picture across the spectrum was scattered, but effect sizes tend to increase with increasing exposure dose. Substantial heterogeneity was also present with respect to all serovar/pathovar subgroups among the included studies. The present body of reports does not display a strong basis for recommendation of relevant intensities, wavelengths and exposure doses for superficial blue light decontamination in medical or food safety contexts. A serious shortcoming in most studies is the absence of a clear documentation of inoculum preparation and of study parameters. We suggest improvement for study protocols for future investigations.

## Introduction

The use of light as an alternative to chemical or thermal inhibition of unwanted microorganisms has received increased attention in medical, food and agricultural, water, and hygiene applications. The germicidal impact of UV-light is well established (Hijnen et al., 2006; Koutchma, 2008, 2018; Hong et al., 2014; Song et al., 2016; Marasini et al., 2021), especially with respect to microbes associated to surfaces. It has been successfully used in controlling water disinfection (e.g., Pullerits et al., 2020), various skin infections (e.g., Abana et al., 2017) as well as surgical site infections (Buonanno et al., 2013). Modification of the wavelength within the UV-band from 256 to 207 nm reduces the negative impact on human cells while maintaining the germicidal effect as stated for methicillin resistant *Staphylococcus aureus* (Buonanno et al., 2013). Despite a high interest in UV-treatment in food contexts to prolong shelf-life and increase food safety when thermal treatment is not an option, legislation varies in different countries. As such, implementation of UV-light and novel food ingredients requires approval in response to various regulations (Koutchma, 2018). Implementation of UV-based disinfection treatment must take occupational environment legislation into account. Given these disadvantages and corroborated by the development and low cost of light-emitting diodes (LED), applications in the visible light spectrum (400–760 nm) have been highlighted (Lui et al., 2016). This is especially true for the blue, green and red spectral bands.

**FIGURE 1:**
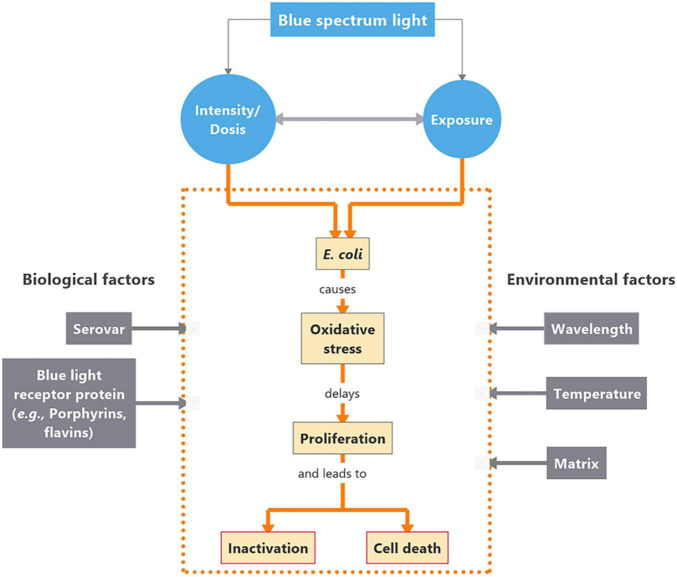
Concept map on interactions between *Escherichia coli* (*E. coli*) and blue light in the short waved band. Intensity of the blue light source as well as the duration of exposure are critical for the impact on the organism’s physiological responses. The cascade of events evolving as a response to blue light exposure are presented within the orange dotted frame. Biological and environmental factors affecting the cascade of events are placed in gray boxes to the left and right side of the box, respectively.

Encouraging results have repeatedly been reported upon photobiological treatment of hazardous microorganisms using blue light (400–500 nm) for different applications, e.g., food safety control (Neal et al., 2012; Guffey et al., 2016; Lacombe et al., 2016; Josewin et al., 2018; Ghate et al., 2019; Hyun and Lee, 2020; Hyun et al., 2021), microbial disease treatment (Plavskii et al., 2018), esp., multidrug resistant strains (Ferrer-Espada et al., 2018), odontology (Cieplik et al., 2014), dermatology (Maclean et al., 2009) or sewage treatment (Giannakis et al., 2015). Some non-phototrophic bacteria are equipped with blue light receptor protein domains (Alsanius et al., 2019 and references therein). The physiological response to blue light varies between different microorganisms and their nutritional environment (Gharaie et al., 2017; Alsanius et al., 2021). Basic functions for microbial spreading and establishment on surfaces may be affected by blue light exposure (Vermeulen et al., 2008; Maclean et al., 2009; Alsanius et al., 2021). Suggested mechanisms involved into the photoinhibitory effect of blue light revolve around the presence of photoreceptor proteins, such as porphyrins or flavins, that can capture a photon converting it into an electronic signaling mechanism (Krauss, 2007). For disinfecting purposes, this signaling mechanism can be used against bacteria through a cascading chain of events ultimately leading to bacterial inhibition or mortality. In the presence of the proper wavelength of light at high enough intensities, Reactive Oxygen Species (ROS) are generated due to this photon capture and conversion, ultimately causing damage to the cell wall (Ryter et al., 2007), DNA, proteins and lipids. Possible photoinhibitory effects are summarized in Figure 1.

In this systematic review, we consider the impact of light quality (i.e., the spectral distribution of light) in the short-waved blue band on *Escherichia coli* (*E. coli*). We focus on the following research questions:

(i) Does short-waved blue light have a deleterious impact on *E. coli*?
(ii) Which wavelengths in the blue spectrum have a deleterious impact on *E. coli*?
(iii) Which intensities cause inactivation or cause lethal effects in *E. coli*?
(iv) Are there any other process parameters that affect inactivation or death of *E. coli* (strain, time, matrix, temperature)?

## Methods

### Systematic Review

The systematic literature review was based on (i) the identification of relevant original research articles using the Scopus database, without time limitation, followed by (ii) data screening and (iii) appointment of included full text articles.

### Data Collection

#### Search Strategy

The literature search was conducted on 15 February, 2021. The search query (Supplementary Table 1) used to identify all relevant peer reviewed articles was (“bluelight” OR “blue-light” OR “blue light”) AND (“*E. coli*” OR “*Escherichia coli*”). No time limitations were set which yielded 430 results.

#### Inclusion and Exclusion Criteria

**FIGURE 2:**
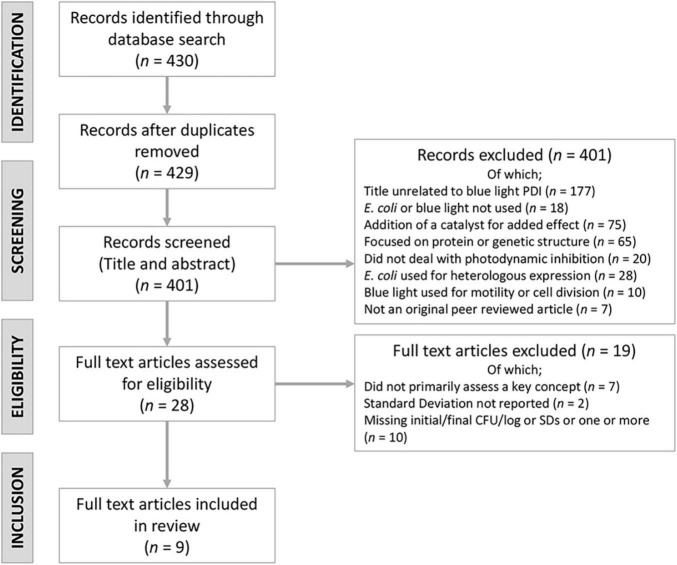
Search criteria and process for selecting articles included in the meta-analysis.

Articles found during the Scopus search were screened and removed or kept based on examination of titles and abstracts. The remaining accepted articles were read for content. Articles were selected based on the flowchart as seen in Figure 2.

#### Inclusion Criteria

Original peer-reviewed articles published in English that met the following criteria were considered for inclusion:

• Included > 1 of the search terms in the title, abstract, or as a keyword (TITLE-ABS-KEY).
• Included serovars and pathovars belonging to *Escherichia coli* (*E. coli*).
• Included wild type or some form of naturally occurring *E. coli* pathovar (not genetically engineered to alter effect or heterologous expressed form).
• Stated specific wavelength as the focal point of light rather than just general “blue light.”
• Assessed for blue light exposure and interactions or its association with inactivation, inhibition, death or efficacy of *E. coli*.
• Reported specific initial and final point of the experiments (CFU or log CFU) with standard deviation for both parameters. Papers reporting just percent inactivation were removed from the final tally.

#### Exclusion Criteria

Articles were excluded if they fulfilled the following criteria;

• Did not primarily assess bacteria-blue light interactions of a specific wavelength or any of the key concepts.
• Did not include serovars and pathovars belonging to *E. coli.*
• Did not deal primarily with the use of blue light for inhibition or inactivation of *E. coli.*
• Were not original peer-reviewed research articles.
• Did not contain sufficient data from *E. coli* experiments to calculate effect sizes.
• Inhibition/inactivation accelerated through the use of a catalyst (not solely due to blue light).
• Paper published in a language other than English with no translation.
• Duplicate hits.

Publications included in the present systematic review are listed in Table 1. It is worthwhile to emphasize that the two publications of Ferrer-Espada and co-workers included into this review used different *E. coli* strains, hence do not constitute any overlapping information.

**TABLE 1:** Included journal articles for this meta-analysis organized by the field of study that used for blue light inhibition of *E. coli*.

Selected Article	Year	Field	Subcategory	Journal
Abana et al.	2016	Medical	MDR Bacteria	Microbiology Open
Ferrer-Espada et al.	2018		MDR Bacteria	Proc. SPIE 10479, Light-Based Diagnosis and Treatment of Infectious Diseases
Ferrer-Espada et al.	2020		General Inhibition	Lasers in Surgery and Medicine
Cieplik et al.	2013	Dental	General Inhibition	Clinical Oral Investigations
Guffey et al.	2016	Food Safety	Food processing	Food Science and Nutrition
Kim and Kang	2021		General Inhibition	Food Research International
Lacombe et al.	2016		Food processing	Journal of Food Protection
Hoenes et al.	2015	Water Safety	Water disinfection	Proc. SPIE 9540, Novel Biophotonics Techniques and Applications III
Plavskii et al.	2018	General Inhibition	General Inhibition	Journal of Photochemistry and Photobiology, B: Biology

#### Data Conversion

For samples that only reported initial and final logarithmic measurements, a simple conversion was made from the data reported to the initial and final CFUs.


(1) $$10^{\log value}=CFUs$$

Phyotodynamic inhibition (PDI) was calculated for all data as a percent decrease in CFUs.


(2) $$PDI\left(\%\right)=100\times \left(1-\frac{CFU^{final}}{CFU^{initial}}\right)$$

For any selected articles reporting only intensity ($\frac{mW}{cm^{2}}$), time (minutes), or exposure dose ($\frac{J}{cm^{2}})$, a conversion was made to include all three elements using the following equation:


(3) $$\frac{J}{cm^{2}}=\frac{mW}{cm^{2}}\times 1000\times \left(minutes\times 60\frac{seconds}{minute}\right)$$

#### Data Analysis

In total, five meta-analyses were performed: (1) of all included studies, to estimate effect of blue light treatment on *E. coli* reduction, (2) subgroup-analysis based on reported intensities, (3) subgroup-analysis based on reported wavelength, (4) subgroup-analysis based on reported exposure dose, and (5) subgroup-analysis based on reported serovar/pathovar. These were conducted using Cochrane’s software for meta-analyses, Review Manager 5.4.1. Random effects models were used consistently to facilitate conclusions applicable beyond the included data (Tufanaru et al., 2015; Higgins et al., 2019). Standardized mean scores were calculated for each included study’s initial and final number of colony-forming units (CFU). Standardized mean differences were used to establish the effect size (Hedges’ g) on CFU reduction after blue light treatment compared to initial CFU for each included study. Effect sizes were weighted by the inverse variance of their study, so that data from studies with better precision had greater impact on the aggregated results of the meta-analysis (Moeyaert et al., 2017). Those inverse variance-weighted effect sizes were then averaged to determine the aggregated effect of blue light treatment on CFU of *E. coli*. In the subgroup-analyses, results from different subgroup defining factors (i.e., intensities/wavelengths/exposure/serovar/pathovar) within the same study were treated as individual studies. As an example, in the study reported by Ferrer-Espada et al. (2018), half of the samples were exposed to blue light treatment with 60 mW/cm^2^ intensity while the other half were exposed to an intensity of 100 mW/cm^2^. Therefore, in meta-analysis (2) the data reported from Ferrer-Espada et al. (2018) were treated as two separate studies; Ferrer-Espada et al. (2018) (60 mW/cm^2^), and Ferrer-Espada et al. (2018) (100 mW/cm^2^).

As recommended by Higgins et al. (2019), statistical heterogeneity was assessed using the I^2^ test, and the strength of heterogeneity was determined by the *p*-value of χ^2^. Specifically, the cut-off for a potential problem with heterogeneity was a *p*-value < 0.1 for χ^2^, while I^2^ of 30–60% was interpreted as “may represent moderate heterogeneity,” 50–90% as “may represent substantial heterogeneity,” and 75–100% as “considerable heterogeneity.”

### Quality Assessment

A quality assessment for five bias domains (domain 1: randomization, D1; domain 2: deviation from intended interventions, D2; domain 3: missing outcome data, D3; domain 4: outcome measurement, D4), selection of reported results, D5) was made as recommended by Higgins et al. (2020). Grading (yes, probably yes, no, probably no) was conducted in two independent screenings using a modified version of the signaling question catalog, and screening outcomes summarized in three judgments: “low risk of bias,” “high risk of bias” and “some concerns.” Criteria underlying the translation of screening results to judgments are listed in Supplementary Table 2.

## Results

**FIGURE 3:**
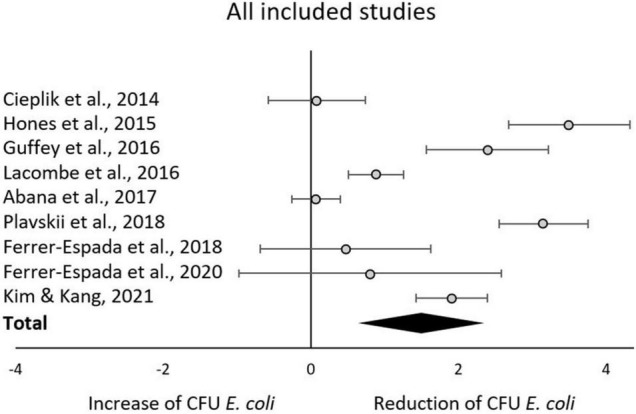
Forest plot of reduction of *E. coli* post blue light exposure in all included studies.

The aggregated results from the initial random effects meta-analysis of all included studies indicated a significant decrease of CFU after blue light treatment. The aggregated effect size (g) was 1.49 (95% confidence interval: 0.64–2.34; *Z* = 3.43, *p* = 0.0006; χ^2^ = 147.19, *p* < 0.00001; I^2^ = 95%). Estimated effects for each included study are presented in Figure 3 and Table 2. As expected, similar aggregated results were found in the subsequent subgroup-analyses.

**TABLE 2:** Characteristics of all included studies.

Study	N	Temp. during exposure (°C)	Wave-length (nm)	Exposure length (min)	Exposure dose (J/cm^2^)	PDI	log (I)	log (K)	log-reduction	Initial mean CFU (SD)	Final mean CFU (SD)	Weight	Effect size (95% CI)
Cieplik et al., 2014	18	25	460	0.33–2	25–150	0.010	7.918	7.914	0.004	8.288E7 (1.028E7)	8.202E7 (1.003E7)	11.6%	0.08
													(−0.57 to 0.74)
Hoenes et al., 2015	30	27	405–460	60–299	120–600	0.821	6.000	5.254	0.746	1.000E6 (2.324E5)	1.793E5 (2.301E5)	11.1%	3.5
													(2.68 to 4.32)
Guffey et al., 2016	20	NR*	405	NR	10–100	0.762	1.215	0.591	0.624	16.400 (5.679)	3.900 (4.500)	11.1%	2.39
													(1.56 to 3.22)
Lacombe et al., 2016	60	NR	405	1–10	0.3–3.3	0.908	6.654	5.618	1.036	4.509E6 (6.456E6)	4.147E5 (1.156E6)	12.1%	0.88
													(0.50 to 1.25)
Abana et al., 2017	72	NR	455	3.8	120	0.225	11.695	11.584	0.111	4.957E11 (1.75E12)	3.840E11 (1.567E12)	12.2%	0.07
													(−0.26 to 0.39)
Plavskii et al., 2018	48	NR	405–445	15–180	45–540	0.675	2.111	1.622	0.488	129.000 (12.400)	41.900 (36.700)	11.7%	3.15
													(2.55 to 3.76)
Ferrer-Espada et al., 2018	6	NR	405	45–96	162–576	0.769	7.577	6.940	0.637	3.779E7 (7.376E7)	8.717E6 (3.166E7)	10.1%	0.47
													(−0.68 to 1.63)
Ferrer-Espada et al., 2020	3	30	405	60	216	0.425	8.260	8.020	0.240	1.820E8 (9.321E7)	1.047E8 (5.620E7)	8.1%	0.8
													(−0.98 to 2.58)
Kim and Kang, 2021	48	NR	395–425	12–81	10–70	0.890	9.346	8.389	0.957	2.217E9 (1.25E9)8	2.447E8 (7.127E8)	11.9%	1.91
													(1.43 to 2.40)
Total	305	25–30	395–460	0.33–299	0.3–600							100%	1.49
													(0.64 to 2.34)

*NR, not recorded.

*PDI, photodynamic inactivation; log(I), pre-treatment log CFU-value; log(K), post-treatment log CFU-value.*

**FIGURE 4:**
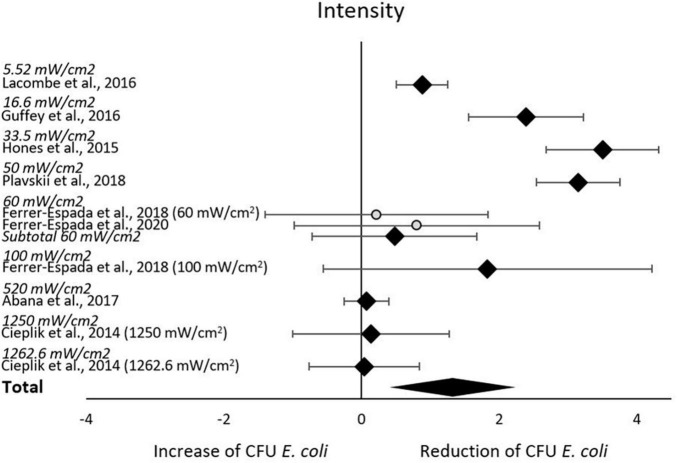
Forest plot of reduction of *E. coli* post blue light exposure by intensity.

The subgroup-analysis of intensities indicated a significant decrease of CFU after blue light treatment for all studies using intensities of 5.52–50 mW/cm^2^, but none of the studies using intensities of 60–1,262.6 mW/cm^2^. Differences in CFU decrease among subgroups were significant (χ^2^ = 135.5, *p* < 0.00001). Tests of statistical heterogeneity for subgroup 60 mW/cm^2^ showed χ^2^ = 0.23, *p* = 0.64; I^2^ = 0%. Tests of statistical heterogeneity were not applicable to the remaining subgroups of this analysis as the amount of studies was insufficient. Statistical heterogeneity for the entire intensities subgroup-analysis was indicated by χ^2^ = 135.71, *p* < 0.00001; I^2^ = 93%. Figure 4 and Table 3 display estimated effects for each included study and subgroup.

**TABLE 3:** CFU *E. coli* outcomes by intensity.

Subgroup	N	PDI	log (I)	log (K)	log-reduction	Initial mean CFU (SD)	Final mean CFU (SD)	Weight	Effect size (95% CI)	Z (*p*-value)
*5.52 mW/cm^2^*										
Lacombe et al., 2016	60	0.908	6.654	5.618	1.036	4.509E6	4.147E5	11.7%	0.88	4.58
						(6.456E6)	(1.156E6)		(0.50 to 1.25)	(<0.00001)
*16.6 mW/cm^2^*										
Guffey et al., 2016	20	0.762	1.215	0.591	0.624	16.4	3.9	10.8%	2.39	5.65
						(5.6791)	(4.5)		(1.56 to 3.22)	(<0.00001)
*33.5 mW/cm^2^*										
Hoenes et al., 2015	30	0.821	6.000	5.254	0.746	1.000E6	1.793E5	10.8%	3.5	8.35
						(2.324E5)	(2.302E5)		(2.68 to 4.32)	(<0.00001)
*50 mW/cm^2^*										
Plavskii et al., 2018	48	0.675	2.111	1.622	0.488	129.000	41.900	10.8%	3.15	10.19
						(12.400)	(36.700)		(2.55 to 3.76)	(<0.00001)
*60 mW/cm^2^*										
Ferrer-Espada et al., 2020	3	0.425	8.260	8.020	0.240	1.820E8	1.047E8	8.0%	0.8	
						(9.321E7)	(5.620E7)		(−0.98 to 2.58)	
Ferrer-Espada et al., 2018 (60 mW/cm^2^)	3	0.583	7.620	7.240	0.380	4.169E7	1.738E7	8.5%	0.22	
						(1.145E8)	(4.775E7)		(−1.39 to 1.84)	
*Subtotal 60 mW/cm^2^*	6							16.6%	0.48	0.79
									(−0.71 to 1.68)	(−0.43)
*100 mW/cm^2^*										
Ferrer-Espada et al., 2018 (100 mW/cm^2^)	3	0.998	7.530	4.750	2.780	3.388E7	5.623E4	6.4%	1.83	1.51
						(2.087E7)	(3.464E4)		(−0.55 to 4.22)	(−0.13)
*520 mW/cm^2^*										
Abana et al., 2017	72	0.225	11.695	11.584	0.111	4.957E11	3.84E11	11.7%	0.07	0.4
						(1.750E12)	(1.5670E12)		(−0.26 to 0.39)	(−0.69)
*1250 mW/cm^2^*										
Cieplik et al., 2014 (1250 mW/cm^2^)	6	0.022	7.918	7.909	0.010	8.288E7	8.102E7	10.0%	0.14	0.25
						(1.094E7)	(1.289E7)		(−0.99 to 1.28)	(−0.8)
*1262.6 mW/cm^2^*										
Cieplik et al., 2014 (1262.6 mW/cm^2^)	12	0.004	7.918	7.917	0.002	8.288E7	8.252E7	10.9%	0.04	0.09
						(1.043E7)	(8.901E6)		(−0.77 to 0.84)	(−0.93)
Total	263							100%	1.32	2.93
									(0.44 to 2.20)	(−0.003)

*PDI, photodynamic inactivation; log(I), pretreatment log CFU-value; log(K), post-treatment log CFU-value.*

**FIGURE 5:**
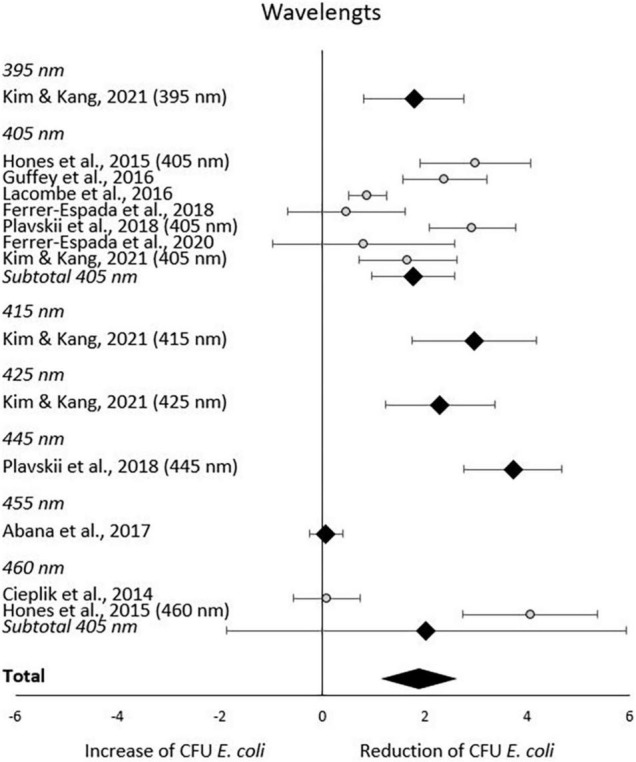
Forest plot of reduction of *E. coli* post blue light exposure by wavelength.

In the subgroup-analysis of wavelengths, significant CFU decrease was seen in subgroups of 395–445 nm but not in subgroups of 455–460 nm. Differences in CFU decrease among subgroups were significant (χ^2^ = 83.6, *p* < 0.00001) Tests of statistical heterogeneity for subgroup 405 nm showed χ^2^ = 37.23, *p* < 0.00001; I^2^ = 84%, and χ^2^ = 28.22, < 0.00001; I^2^ = 96% for subgroup 460 nm. Tests of statistical heterogeneity were not applicable to the remaining subgroups of this analysis as the amount of studies was insufficient. Tests of statistical heterogeneity for the entire wavelengths subgroup-analysis showed χ^2^ = 155.03, *p* < 0.00001; I^2^ = 92%. Figure 5 and Table 4 display estimated effects for each included study and subgroup.

**TABLE 4:** CFU *E. coli* outcomes by wavelength.

Subgroup	N	PDI	log (I)	log (K)	log-reduction	Initial mean CFU (SD)	Final mean CFU (SD)	Weight	Effect size (95% CI)	Z (*p*-value)
*395 nm*										
Kim and Kang, 2021 (395 nm)	12	0.773	9.178	8.535	0.643	1.508E9	3.430E8	7.2%	1.79	3.6
						(5.958E8)	(6.615E7)		(0.81 to 2.76)	0.0003
*405 nm*										
Ferrer-Espada et al., 2018	6	0.769	7.577	6.940	0.637	3.779E7	8.717E6	6.8%	0.47	
						(7.376E7)	(3.165E7)		(−0.68 to 1.63)	
Ferrer-Espada et al., 2020	3	0.425	8.260	8.020	0.240	1.820E8	1.047E8	5.5%	0.8	
						(9.321E7)	(5.620E7)		(−0.98 to 2.58)	
Guffey et al., 2016	20	0.762	1.215	0.591	0.624	16.4	3.9	7.5%	2.39	
						(5.6791)	(4.5)		(1.56 to 3.22)	
Lacombe et al., 2016	60	0.908	6.654	5.618	1.036	4.510E6	4.147E5	8.1%	0.88	
						(6.456E6)	(1.156E6)		(0.50 to 1.25)	
Hoenes et al., 2015 (405 nm)	15	0.748	6.000	5.402	0.598	1.000E6	2.524E5	7.0%	2.99	
						(2.365E5)	(2.502E5)		(1.91 to 4.07)	
Plavskii et al., 2018 (405 nm)	24	0.740	2.111	1.526	0.584	129	33.6	7.5%	2.93	
						(12.5)	(43.6)		(2.09 to 3.76)	
Kim and Kang, 2021 (405 nm)	12	0.895	9.277	8.300	0.977	1.894E9	1.997E8	7.2%	1.67	
						(7.605E8)	(1.159E8)		(0.72 to 2.62)	
*Subtotal 405 nm*	*140*							*49.5%*	*1.77*	*4.27*
										(<0.0001)
*415 nm*										
Kim and Kang, 2021 (415 nm)	12	0.920	9.554	8.456	1.098	3.584E9	2.860E8	6.7%	2.96	4.77
						(1.421E9)	(5.398E8)		(1.75 to 4.18)	(<0.00001)
*425 nm*										
Kim and Kang, 2021 (425 nm)	12	0.920	9.274	8.176	1.098	1.880E9	1.501E8	7.0%	2.3	4.21
						(9.902E8)	(2.722E8)		(1.23 to 3.37)	(<0.0001)
*445 nm*										
Plavskii et al., 2018 (445 nm)	24	0.610	2.111	1.702	0.409	129	50.3	7.2%	3.72	7.59
						(12.5)	(26.6)		(2.76 to 4.68)	(<0.00001)
*455 nm*										
Abana et al., 2017	72	0.225	11.695	11.584	0.111	4.957E11	3.840E11	8.2%	0.07	0.4
						(1.750E12)	(1.567E12)		(−0.26 to 0.39)	−0.69
*460 nm*										
Cieplik et al., 2014	18	0.010	7.918	7.914	0.004	8.288E7	8.202E7	7.8%	0.08	
						(1.028E7)	10031536.4		(−0.57 to 0.74)	
Hoenes et al., 2015 (460 nm)	15	0.894	6.000	5.026	0.974	1.000E6	1.063E5	6.5%	4.06	
						(2.365E5)	(1.891E5)		(2.75 to 5.38)	
*Subtotal 460 nm*	33							14.2%	2.03	1.02
									(−1.87 to 5.93)	−0.31
Total	305							100%	1.9	5.28
									(1.20 to 2.61)	(<0.00001)

*PDI, photodynamic inactivation; log(I), pretreatment log CFU-value; log(K), post-treatment log CFU-value.*

**FIGURE 6:**
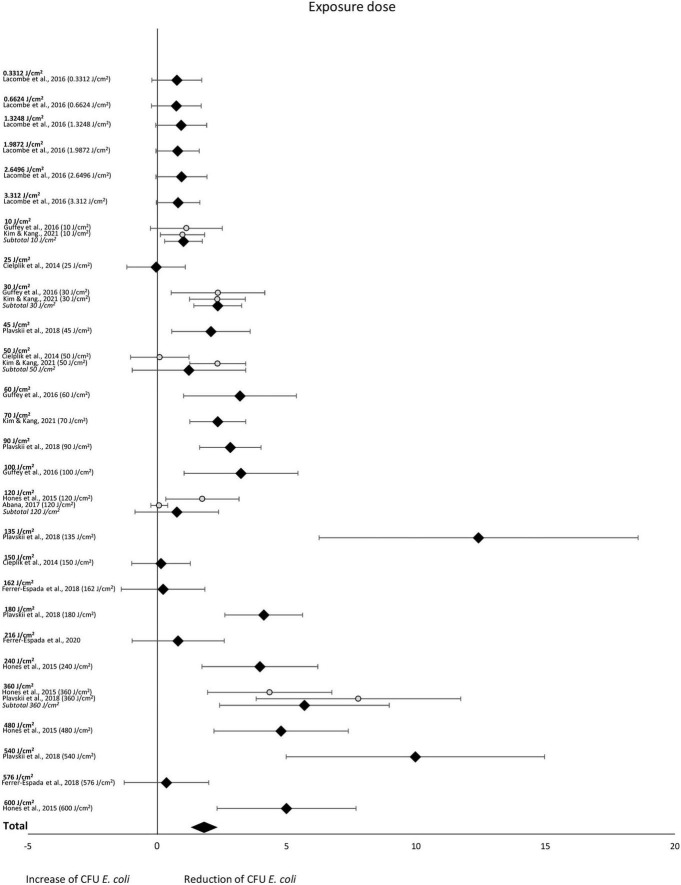
Forest plot of reduction of *E. coli* post blue light exposure by exposure dose.

The subgroup-analysis of exposure dose indicated scattered results, where a significant CFU decrease was seen among subgroups of exposure dose 10, 30, 45, 60, 70, 90, 100, 135, 180, 240, 360, 480 and 540 J/cm^2^ but not among subgroups of exposure dose 0.3312, 0.6624, 1.3248, 1.9872, 2.6496, 3.312, 25, 50, 120, 150, 162, 216 or 576 J/cm^2^. Differences in CFU decrease among subgroups were significant (χ^2^ = 105.5, *p* < 0.00001). Tests of statistical heterogeneity showed following results: χ^2^ = 0.03, *p* = 0.85; I^2^ = 0% for subgroup 10 J/cm^2^; χ^2^ = 0.00, *p* = 0.99; I^2^ = 0% for subgroup 30 J/cm^2^; χ^2^ = 7.87, *p* = 0.005; I^2^ = 87% for subgroup 50 J/cm^2^; χ^2^ = 5.09, *p* = 0.02; I^2^ = 80% for subgroup 180 J/cm^2^; χ^2^ = 2.11, *p* = 0.15; I^2^ = 53% for subgroup 360 J/cm^2^. Tests of statistical heterogeneity were not applicable to the remaining subgroups of this analysis as the amount of studies was insufficient. Tests of statistical heterogeneity for the entire exposure dose subgroup-analysis showed χ^2^ = 165.25, *p* < 0.00001; I^2^ = 81%. Figure 6 and Table 5 display estimated effects for each included study and subgroup.

**TABLE 5:** CFU *E. coli* outcomes by exposure doses.

Subgroup	N	PDI	log (I)	log (K)	log-reduction	Initial mean CFU (SD)	Final mean CFU (SD)	Weight	Effect size (95% CI)	Z (*p*-value)
*0.3312 J/cm^2^*										
Lacombe et al., 2016 (0.3312 J/cm^2^)	9	0.791	6.699	6.020	0.679	5.003E6	1.048E6	3.9%	0.75	1.52
						(6.963E6)	(1.596E6)		(−0.22 to 1.71)	(0.13)
*0.6624 J/cm^2^*										
Lacombe et al., 2016 (0.6624 J/cm^2^)	9	0.798	6.699	6.005	0.695	5.003E6	1.011E6	3.9%	0.73	1.49
						(6.963E6)	(2.394E6)		(−0.23 to 1.69)	(0.14)
*1.3248 J/cm^2^*										
Lacombe et al., 2016 (1.3248 J/cm^2^)	9	0.948	6.699	5.415	1.284	5.003E6	2.599E5	3.9%	0.92	1.83
						(6.963E6)	(3.439E5)		(−0.07 to 1.90)	(0.07)
*1.9872 J/cm^2^*										
Lacombe et al., 2016 (1.9872 J/cm^2^)	12	0.959	6.576	5.188	1.388	3.768E6	1.543E5	4.1%	0.78	1.82
						(6.344E6)	(2.766E5)		(−0.06 to 1.61)	(0.07)
*2.6496 J/cm^2^*										
Lacombe et al., 2016 (2.6496 J/cm^2^)	9	0.966	6.699	5.227	1.472	5.003E6	1.685E5	3.9%	0.93	1.86
						(6.963E6)	(3.231E5)		(−0.05 to 1.92)	(0.06)
*3.312 J/cm^2^*										
Lacombe et al., 2016 (3.312 J/cm^2^)	12	0.986	6.576	4.734	1.842	3.768E6	5.416E4	4.1%	0.80	1.87
						(6.344E6)	(8.924E4)		(−0.04 to 1.64)	(0.06)
*10 J/cm^2^*										
Guffey et al., 2016 (10 J/cm^2^)	5	0.378	1.215	1.009	0.206	16.4	10.2	3.4%	1.12	
						(6.2)	(3.4)		(−0.27 to 2.51)	
Kim and Kang, 2021 (10 J/cm^2^)	12	0.560	9.346	8.989	0.357	2.217E9	9.747E8	4.0%	0.97	
						(1.300E9)	(1.181E9)		(0.11 to 1.82)	
*Subtotal 10 J/cm^2^*	17							7.4%	1.01	2.71
									(0.28 to 1.74)	(0.007)
*25 J/cm^2^*										
Cieplik et al., 2014 (25 J/cm^2^)	6	−0.005	7.918	7.921	−0.002	8.288E7	8.332E7	3.7%	−0.05	0.08
						(1.094E7)	(4.943E6)		(−1.18 to 1.08)	(0.93)
*30 J/cm^2^*										
Guffey et al., 2016 (30 J/cm^2^)	5	0.756	1.215	0.602	0.613	16.4	4.0	2.9%	2.34	
						(6.2)	(2.7)		(0.53 to 4.15)	
Kim and Kang, 2021 (30 J/cm^2^)	12	0.998	9.346	6.608	2.738	2.217E9	4.051E6	3.8%	2.32	
						(1.300E9)	(1.440E7)		(1.25 to 3.40)	
*Subtotal 30 J/cm^2^*	17							6.7%	2.33	4.94
									(1.40 to 3.25)	(<0.00001)
*45 J/cm^2^*										
Plavskii et al., 2018 (45 J/cm^2^)	6	0.190	2.111	2.019	0.091	129.0	104.5	3.2%	2.07	2.67
						(13.4)	(7.7)		(0.55 to 3.58)	(0.008)
*50 J/cm^2^*										
Cieplik et al., 2014 (50 J/cm^2^)	6	0.014	7.918	7.912	0.006	8.288E7	8.173E7	3.7%	0.09	
						(1.094E7)	(1.218E7)		(−1.04 to 1.22)	
Kim and Kang, 2021 (50 J/cm^2^)	12	1.000	9.346	4.108	5.238	2.217E9	1.281E4	3.8%	2.33	
						(1.300E9)	(2.694E4)		(1.25 to 3.41)	
*Subtotal 50 J/cm^2^*	18							7.5%	1.22	1.09
									(−0.98 to 3.41)	(0.28)
*60 J/cm^2^*										
Guffey et al., 2016 (60 J/cm^2^)	5	0.951	1.215	−0.097	1.312	16.4	0.8	2.5%	3.19	2.87
						(6.2)	(0.8)		(1.01 to 5.37)	(0.004)
*70 J/cm^2^*										
Kim and Kang, 2021 (70 J/cm^2^)	12	1.000	9.346	2.887	6.459	2.217E9	771.2	3.8%	2.33	4.24
						(1.230E9)	(876.9)		(1.25 to 3.41)	(<0.0001)
*90 J/cm^2^*										
Plavskii et al., 2018 (90 J/cm^2^)	12	0.550	2.111	1.764	0.346	129.0	58.1	3.7%	2.82	4.67
						(12.8)	(31.8)		(1.64 to 4.01)	(<0.00001)
*100 J/cm^2^*										
Guffey et al., 2016 (100 J/cm^2^)	5	0.963	1.215	−0.222	1.437	16.4	0.6	2.4%	3.23	2.88
						(6.2)	(0.9)		(1.03 to 5.43)	(0.004)
*120 J/cm^2^*										
Abana et al., 2017	72	0.225	11.695	11.584	0.111	4.957E11	3.84E11	4.5%	0.07	
						(1.75E12)	(1.567E12)		(−0.26 to 0.39)	
Hoenes et al., 2015 (120 J/cm^2^)	6	0.439	6.000	5.749	0.251	1.000E6	5.606E5	3.4%	1.74	
						(2.503E5)	(2.154E5)		(0.32 to 3.15)	
*Subtotal 120 J/cm^2^*	78							7.9%	0.75	0.92
									(−0.86 to 2.37)	(0.36)
*135 J/cm^2^*										
Plavskii et al., 2018 (135 J/cm^2^)	6	0.990	2.111	0.114	1.997	129.0	1.3	0.6%	12.41	3.95
						(13.4)	(0.5)		(6.24 to 18.57)	(<0.0001)
*150 J/cm^2^*										
Cieplik et al., 2014 (150 J/cm^2^)	6	0.022	7.918	7.909	0.010	8.288E7	8.102E7	3.7%	0.14	0.25
						(1.094E7)	(1.289E7)		(−0.99 to 1.28)	(0.80)
*162 J/cm^2^*										
Ferrer-Espada et al., 2018 (162 J/cm^2^)	3	0.583	7.620	7.240	0.380	4.169E7	1.738E7	3.1%	0.22	0.27
						(1.145E8)	(4.775E7)		(−1.39 to 1.84)	(0.79)
*180 J/cm^2^*										
Plavskii et al., 2018 (180 J/cm^2^)	12	0.775	2.111	1.462	0.648	129.0	29.0	3.3%	4.11	5.36
						(12.8)	(30.6)		(2.61 to 5.62)	(<0.00001)
*216 J/cm^2^*										
Ferrer-Espada et al., 2020	3	0.425	8.26	8.02	0.240	1.820E8	1.0471E8	2.9%	0.80	0.89
						(9.321E7)	(5.620E7)		(−0.98 to 2.58)	(0.38)
*240 J/cm^2^*										
Hoenes et al., 2015 (240 J/cm^2^)	6	0.844	6	5.194	0.806	1.000E6	1.562E5	2.4%	3.96	3.46
						(2.503E5)	(1.217E5)		(1.72 to 6.20)	(0.0005)
*360 J/cm^2^*										
Hoenes et al., 2015 (360 J/cm^2^)	6	0.907	6	4.969	1.030	1.000E6	9.330E4	2.2%	4.34	
						(2.503E5)	(1.089E5)		(1.94 to 6.74)	
Plavskii et al., 2018 (360 J/cm^2^)	6	0.760	2.111	1.491	0.619	129.0	31.0	1.2%	7.77	
						(13.4)	(9.5)		(3.81 to 11.72)	
*Subtotal 360 J/cm^2^*	12							3.4%	5.68	3.39
									(2.40 to 8.96)	(0.0007)
*480 J/cm^2^*										
Hoenes et al., 2015 (480 J/cm^2^)	6	0.946	6	4.730	1.270	1.000E6	5.367E4	2.1%	4.78	3.61
						(2.503E5)	(6.501E4)		(2.18 to 7.37)	(0.0003)
*540 J/cm^2^*										
Plavskii et al., 2018 (540 J/cm^2^)	6	0.810	2.111	1.389	0.721	129.0	24.5	0.8%	9.97	3.91
						(13.4)	(2.6)		(4.97 to 14.96)	(<0.0001)
*576 J/cm^2^*										
Ferrer-Espada et al., 2018 (576 J/cm^2^)	3	0.998	7.53	4.750	2.780	3.388E7	5.623E4	3.1%	0.35	0.42
						(9.321E7)	(5.620E7)		(−1.28 to 1.99)	(0.67)
*600 J/cm^2^*										
Hoenes et al., 2015 (600 J/cm^2^)	6	0.967	6	4.517	1.483	1.000E6	3.287E4	2.0%	4.99	3.64
						(2.503E5)	(3.803E4)		(2.30 to 7.67)	(0.0003)
Total	305							100%	1.81	7.05
									(1.31 to 2.32)	(<0.00001)

*PDI, photodynamic inactivation; log(I), pretreatment log CFU-value; log(K), post-treatment log CFU-value.*

**FIGURE 7:**
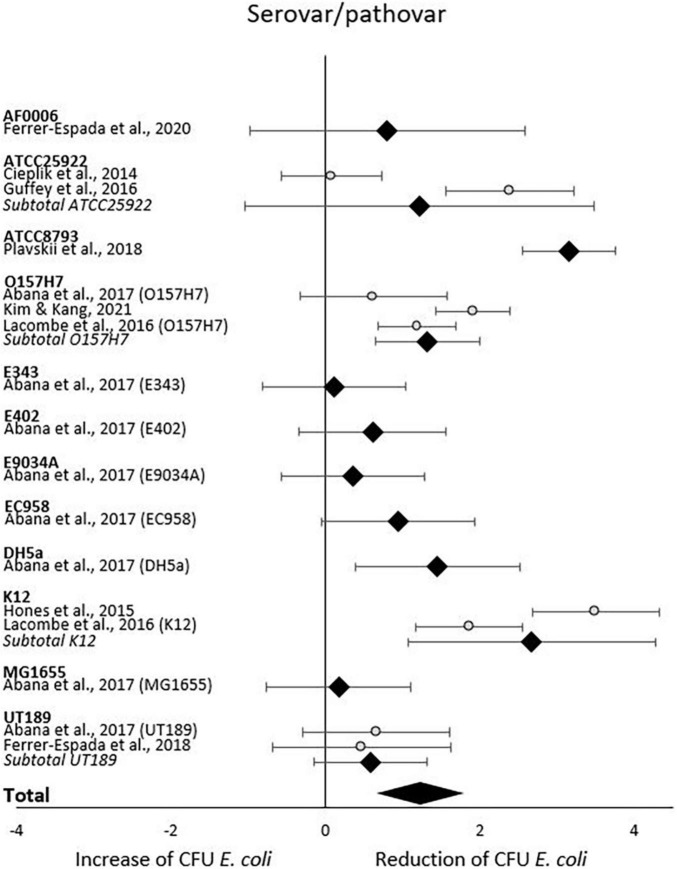
Forest plot of reduction of *E. coli* post blue light exposure by serovar/pathovar.

When analyzing initial and final CFU based on serovar/pathovar in the last subgroup-analysis, significant CFU decrease was seen for ATCC8793, O157H7, DH5a and K12 but not for AF0006, ATCC 25922, E343, E402, E9034A, EC958, MG1655 or UT189. Differences in CFU decrease among subgroups were significant (χ^2^ = 61.1, *p* < 0.00001). Tests of statistical heterogeneity showed following results: χ^2^ = 5.65, *p* = 0.02; I^2^ = 82% for subgroup O157H7; χ^2^ = 0.06, *p* = 0.81; I^2^ = 0% for subgroup UT189; χ^2^ = 18.37, *p* = 0.0001; I^2^ = 95% for subgroup ATCC 25922; χ^2^ = 9.02, *p* = 0.003; I^2^ = 89% for subgroup K12. Tests of statistical heterogeneity were not applicable to the remaining subgroups of this analysis as the amount of studies was insufficient. Tests of statistical heterogeneity for the entire serovar/pathovar subgroup-analysis showed χ^2^ = 114.39, *p* < 0.00001; I^2^ = 87%. Figure 7 and Table 6 display estimated effects for each included study and subgroup.

**TABLE 6:** CFU *E. coli* outcomes by serovar/pathovar.

Subgroup	N	PDI	log (I)	log (K)	log-reduction	Initial mean CFU (SD)	Final mean CFU (SD)	Weight	Effect size (95% CI)	Z (*p*-value)
*MG1655*										
Abana et al., 2017 (MG1655)	9	0.184	12.558	12.470	0.088	3.618E + 12	2.954E12	6.3%	0.17	0.36
						(3.814E12)	(3.647E12)		(−0.76 to 1.10)	(0.72)
*E343*										
Abana et al., 2017 (E343)	9	0.085	9.711	9.673	0.038	5.145E9	4.711E9	6.3%	0.11	0.23
						(4.587E9)	(2.739E9)		(−0.82 to 1.03)	(0.82)
*E402*										
Abana et al., 2017 (E402)	9	0.292	9.751	9.601	0.150	5.633E9	3.986E9	6.2%	0.61	1.26
						2.838E9	(2.278E9)		(−0.34 to 1.56)	(0.21)
*O157H7*										
Kim and Kang, 2021	48	0.890	9.346	8.389	0.957	2.217E9	2.447E8	6.7%	1.91	
						(1.258E9)	(7.127E8)		(1.43 to 2.40)	
Abana et al., 2017 (OH157H7)	9	0.686	11.129	10.626	0.502	1.345E11	4.230E10	5.8%	0.62	
						(1.962E11)	(4.392E10)		(−0.33 to 1.57)	
Lacombe et al., 2016 (OH157H7)	36	0.917	5.780	4.698	1.082	6.024E5	4.985E4	6.7%	1.19	
						(6.466E4)	(8.335E4)		(0.68 to 1.69)	
*Subtotal O157H7*	93							19.2%	1.32	2.07
									(0.64 to 2.00)	(0.04)
*E9034A*										
Abana et al., 2017 (E9034A)	9	0.208	9.782	9.681	0.102	6.056E9	4.793999481E9	5.8%	0.36	0.76
						(3.415E9)	(3.255E9)		(−0.57 to 1.29)	(0.45)
*UT189*										
Ferrer-Espada et al., 2018	6	0.769	7.577	6.940	0.637	3.779E7	8.717E6	5.3%	0.47	
						(7.376E7)	(3.166E7)		(−0.68 to 1.63)	
Abana et al., 2017 (UT189)	9	0.727	11.182	10.619	0.564	1.521E11	4.155E10	5.8%	0.66	
						(2.19E11)	(5.715E10)		(−0.30 to 1.61)	
*Subtotal UT189*	15							11.1%	0.58	1.55
									(−0.15 to 1.32)	(0.12)
*EC958*										
Abana et al., 2017 (EC958)	9	0.519	10.633	10.315	0.318	4.299E10	2.067E10	5.7%	0.94	1.86
						(3.013E10)	(1.111E10)		(−0.05 to 1.92)	(0.06)
*ATCC 25922*										
Guffey et al., 2016	20	0.762	1.215	0.591	0.624	16.4	3.9	6.0%	2.39	
						(5.7)	(4.5)		(1.56 to 3.22)	
Cieplik et al., 2014	18	0.010	7.918	7.914	0.004	8.288E7	8.202E7	6.4%	0.08	
						(1.028E7)	(1.003E7)		(−0.57 to 0.74)	
*Subtotal ATCC 25922*	38							12.5%	1.22	1.06
									(−1.04 to 3.49)	(0.29)
*AF0006*										
Ferrer-Espada et al., 2020	3	0.425	8.260	8.020	0.240	1.820E8	1.047128548E8	3.9%	0.80	0.89
						(9.321E7)	(5.620E7)		(−0.98 to 2.58)	(0.38)
*K12*										
Hoenes et al., 2015	30	0.821	6.000	5.254	0.746	1.000E6	1.793E5	6.1%	3.50	
						(2.324E5)	(2.302E5)		(2.68 to 4.32)	
Lacombe et al., 2016 (K12)	24	0.907	7.016	5.983	1.033	1.037E7	9.621E5	6.4%	1.86	
						(6.823E6)	(1.703E6)		(1.17 to 2.55)	
*Subtotal K12*	54							12.4%	2.67	3.25
									(1.06 to 4.27)	(0.001)
*ATCC 8793*										
Plavskii et al., 2018	48	0.675	2.111	1.622	0.488	129.0	41.9	6.5%	3.15	10.19
						(12.4)	(36.7)		(2.55 to 3.76)	(0.00001)
*DH5a*										
Abana et al., 2017 (DH5a)	9	0.981	9.221	7.490	1.732	1.665E9	3.089E7	5.5%	1.45	2.66
						(1.521E9)	(4.689E7)		(0.38 to 2.51)	(0.008)
Total	305							100%	1.23	4.15
									(0.70 to 1.75)	(<0.0001)

*PDI, photodynamic inactivation; log(I), pretreatment log CFU-value; log(K), post-treatment log CFU-value.*

## Quality Assessment and Bias of Risks

**FIGURE 8:**
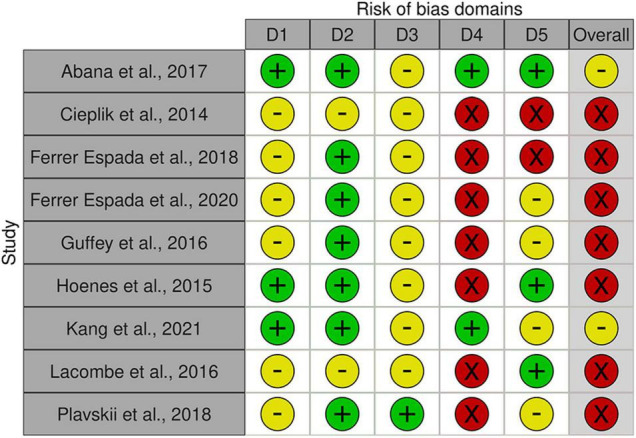
Risk of bias assessment using traffic light plots and domain level judgments for each study using the Robvis-tool [Risk of bias tools—robvis (visualization tool)]. Five domains were considered based on Higgins et al. (2020) (D1: bias to randomization; D2: Bias due to deviations from intended interventions; D3: Bias due to missing data; D4: Bias due to outcome measurement; D5: Bias to selection of reported results). Judgments are based on multiple criteria and marked as + in green field: low risk,—in yellow field: some concerns and—in red field: high risk (see Supplementary Table 2).

Apart from the broad diversity of light intensities and exposure as well as *E. coli* strains, a range of matrices were used in the present set of studies, complicating comparability between studies. The overall outcome quality assessment of bias indicated in general some concerns or high risk of bias across studies (Figure 8). In detail, none of the studies was distinct with respect to randomization of samples during preparation, exposure, nor during analysis (order of sampling, enumeration; domain 1 “Randomization,” D1). An additional confounding factor was the absence of standardization procedures with respect to the physiological status of the inoculum (exceptions: Abana et al., 2017; Kim and Kang, 2021) or ray distribution (exception: Hoenes et al., 2015). No deviations from intended interventions or withdrawal of information were observed (Domain 2, D2). In this context it is worthwhile to note, that the studies only report on sample numbers included into the statistical analysis and thus do not allow a clear judgment. Likewise, with respect to domain 3, missing outcome data (D3), the assessment was left with uncertainties. Multiple concerns became evident with respect to the measurement of the outcome (domain 4, D4). Most studies based their analysis on viable count. Upon stress, cells of some microbial species may transcend to different forms of dormancy, e.g., viable but not culturable (VBNC), persister cells. This has also been demonstrated for *E. coli*. Only two of the involved studies (Abana et al., 2017; Kim and Kang, 2021) account for the dilemma evolving from the exclusiveness in viability concepts, but these studies used different methods to assess viability. There are several additional issues regarding measurement—namely absence of true control treatments (unexposed samples) as well as uncertainties regarding abiotic experimental conditions (i.e., temperature) and exposure length. In combination, they may provoke a crucial mass of biases. (i) the interaction between microbial growth and temperature is well known. Temperature regime during inoculum propagation and during blue light incubation influences cell viability. (ii) Exposure is a function of light intensity and time (Equation 3). Exposure length may be used to compensate for light sources with lower intensity. Depending on the strength of the light source, the additional exposure length may be substantial and additional microbial propagation may take place. It is thus critical that true controls are incorporated into such experiments, allowing standardization of potential additional growth. The present set of studies used the microbial titer before exposure to blue light as the reference to compare the outcome of the treatment. This complex of biases needs to be viewed in the light of lacking reports on experimental conditions and cell age. Last, but not least, concerns with respect to the selection of reported results (domain 5, D5) need attention, as few studies distinctly document or involve enough true replicates, technical replicates and repetitions of the interventions. Ultimately, no studies were selected for removal due to bias because any potential discriminating factors were widespread throughout studies on blue light decontamination of *E. coli*.

## Discussion

There is a substantial interest from medical, food and environmental sciences on short waved blue light to circumvent the use of antibiotics to treat bacterial skin and oral infections, to increase food safety, and to aid in environmental remediation (e.g., sewage water). Blue light often appears to be the new silver bullet. Indeed, this systematic review supports the reducing potential by the short wave blue light of culturable *E. coli* (Figure 3). From the included body of literature, both light intensity and wavelength were identified as the most decisive factors to reduce the number of culturable *E. coli.* Despite the significantly large effect size, considerable heterogeneity was present across studies, meaning that the effect of blue light treatment is not uniform. The impact of light intensities and wavelengths on the occurrence of culturable *E. coli* ceased when exceeding 50 mW/cm^2^ and 445 nm, respectively. However, caution is required when interpreting these results. For example, only the intensity subgroup employing 60 mW/cm^2^ consisted of more than one study. A light intensity of 50 mW/cm^2^ might be considered as a possible cut-off value. However, the considerable heterogeneity across all studies blurs the picture and significant results were obtained for some but not all subgroups. The same applies to the subgroup analysis with respect to wavelengths. The existing body of information does not allow any recommendations on suitable intensity, dose or exposure of blue light toward *E. coli* for sanitation or cure, as the number of eligible studies is low and many studies.

• lack sufficient proof on the effect of the treatment on the target organism (viable, but not culturable, VBNC; inactivated; lethal).
• are short in or lack vital information on experimental procedures.

There is a considerable weakness to the mode in which the inactivation of *E. coli* was assessed in the included studies. In all studies, viable count through enumeration of colony-forming units was used. This is a well-established method to estimate the presence of bacteria and bacterial groups which can grow on stationary microbiological media, but there are inherent pitfalls to this method. For the interpretation of the given information, the detection limit needs to be defined. “No growth” on stationary medium under standardized conditions is often interpreted as “absence.” Apart from their potential presence below the detection limit, cells might be present as viable but non-culturable cells (VBNC) and persister cells. The transition to the viable but not culturable stage has been shown for several organisms, including *E. coli* (Oliver, 2010). Upon hostile environmental conditions, cells thus switch to a low, but measurable metabolic stage with maintained respiration and cell wall integrity, but can retain virulence and infectivity upon resuscitation (Oliver, 2010). Bacterial subpopulations entering a dormant, non-dividing stage upon environmental stress (e.g., chemotherapy) are called persister cells. They are known to survive conditions lethal to regular cells (Lewis, 2010). Therefore, revitalization might occur as shown in Giannakis et al. (2015). All three post exposure options listed here lead to recolonization or recurrent infection. The included body of studies most likely underestimate the number of living cells and thus the level of inactivation. RNA-based approaches involving RT-PCR or ddPCR might give a more distinct result on the blue light impact on *E. coli*.

Information on the cell culture’s physiological status is vital and might account for some of the conflicting or scattered results. As demonstrated by Abana et al. (2017) sensitivity to blue light varies between different physiological stages (exponential, transition, stationary). For four out of eight tested strains, the strongest sensitivity to blue light exposure occurred during the exponential phase, while 25% of the tested strains displayed strongest sensitivity during stationary phase when compared to the unexposed controls. Likewise, the mode of growth (planktonic, biofilm) needs to be taken into account, as marginal blue light impact was observed for biofilm associated *E. coli* (Ferrer-Espada et al., 2018, 2020). Thus display of cardinal growth parameters (e.g., generation time) and tested physiological stage for tested strains, their mode of growth as well as a robust operation parameters during propagation and exposure must be included to find relevant PDI levels.

### Strengths and Limitations

This systematic review indicates that the results must be considered with caution (i) as only a small number of studies were included, (ii) as study parameters were very heterogeneous and (iii) as the included studies lacked a robust presentation of study conditions. Although, the study still indicates an inhibitory impact on culturable *E. coli* following blue light exposure, the initial questions regarding threshold levels for wavelength, light intensities and other process parameters affecting photodynamic inactivation remain unanswered.

To disentangle the true picture of the photodynamic inactivation of *E. coli* using blue light, studies would benefit from more basic information about the preculture and culture conditions. Only three of the included studies (Cieplik et al., 2014; Hoenes et al., 2015; Ferrer-Espada et al., 2020) reported the incubation temperature while exhibited to blue light, and information on preculture incubation temperature and lengths as well as nutritional conditions were scarce. Hence, did the studies display the true effect of blue light or rather a mixed impact blurred by the preculture history? Display of growth curves or growth curve parameters during preculture and blue light exhibition would allow for inferences on the physiological stage of the studied cells. This is of premier importance when elaborating on exposure dose. The exposure dose is a measure of the absorbed radiation dose. Depending on the intensity of the chosen LED device, the length of exposure may vary between different studies and thus allow propagation of the cells and thereby contribute to a heterogeneous impact on measured CFU. Long exposure of *E. coli* to low intensities of blue light may also contribute to nutrient depletion upon propagation; obtained results might hide cross stress responses.

The absence of true control groups, following the propagation of *E. coli* under comparable study conditions in the absence of blue light is a major dilemma and contributes considerably to uncertainty. The study design governing the included studies compares viable counts of *E. coli* before and after exposure to blue light, but a control group is not considered. This first named scenario might overestimate the impact of the blue light treatment (or potentially these studies underestimate effects).

### Recommendation

Our analysis shows that *E. coli* may be photodynamically inhibited using blue light. Based on the selected studies and the high level of heterogeneity, it does not display a strong basis for recommendation of relevant intensities, wavelengths or exposure doses for superficial blue light decontamination in medical or food safety contexts. More studies are needed to draw conclusions regarding subgroup parameters, such as exposure, intensity, wavelength and strain. To unravel biases, we strongly recommend requesting future reports to include:

• Preculture and culture conditions: strain (incl. presence of blue light receptor proteins), incubation temperature and length, nutritional conditions and preculture conditions that reflect the study conditions during exposure.
• Experimental design: a true control group/s, methods to evaluate treatment responses that reflect the intended goal of the study (inactivation, inhibition, death) and purpose, methods that avoid long incubation times to compensate for low light intensities, growth curves and growth curve parameters.
• Experimental management: survey of true light intensities as devices might age; survey light transmission through the lid covering the bacterial culture during the experiment.

## Conclusion

1) Exposure to blue light seemingly has a significant and large reducing effect on viable counts of *E. coli.*
2) There is substantial heterogeneity across studies.
3) Among subgroups intensity and wavelength showed the clearest impact.
  a.) Intensity
  b.) Wavelength

4) Exposure dose shows a scattered picture across the spectra, but effect sizes tend to increase with increasing exposure dose.
5) Heterogeneity occurred in all serovar/pathovar subgroups based on the present range of studies.
6) A clear documentation of inoculum preparation and study parameters is mostly absent.
7) We suggest improvement for study protocols for future investigations.

## Author Contributions

CL, SW, and BA: conceptualization, methodology, validation, formal analysis, investigation, writing—original draft preparation, writing—review and editing, and visualization. BA: resources, project administration, and funding acquisition. CL: data curation. All authors contributed to the article and approved the submitted version.

## Conflict of Interest

The authors declare that the research was conducted in the absence of any commercial or financial relationships that could be construed as a potential conflict of interest.

## Publisher’s Note

All claims expressed in this article are solely those of the authors and do not necessarily represent those of their affiliated organizations, or those of the publisher, the editors and the reviewers. Any product that may be evaluated in this article, or claim that may be made by its manufacturer, is not guaranteed or endorsed by the publisher.

## References

[B1] Abana C. M. Brannon J. R. Ebbott R. A. Dunigan T. L. Guckes K. R. Fuseini H. (2017). Characterization of blue light irradiation effects on pathogenic and nonpathogenic *Escherichia coli*. *Microbiol. Open* 6:e466. 10.1002/mbo3.466 28332311 PMC5552948

[B2] Alsanius B. W. Karlsson M. E. Rosberg A. K. Dorais M. Naznin T. Khalil S. (2019). Light and microbial lifestyle: The impact of light quality on plant-microbe interactions in horticultural production systems - a review. *Horticulturae* 5:41. 10.3390/horticulturae5020041

[B3] Alsanius B. W. Vaas L. A. I. Gharaie S. Karlsson M. E. Rosberg A. K. Wohanka W. (2021). Dining in blue light impairs the appetite of some leaf epiphytes. *Front. Microbiol.* 12:725021. 10.3389/fmicb.2021.725021 34733247 PMC8558677

[B4] Buonanno M. Randers-Pehrson G. Bigelow A. W. Trivedi S. Lowy F. D. Spotnitz H. M. (2013). 207-nm UV light - A promising tool for safe low-cost reduction of surgical site infections. I: *In vitro* studie. *PLoS One* 8:e76968. 10.1371/journal.pone.0076968 24146947 PMC3797730

[B5] Cieplik F. Späth A. Leibl C. Gollmer A. Regensburger J. Tabenski L. (2014). Blue light kills Aggregatibacter actinomycetemcomitans due to its endogenous photosensitizers. *Clin. Oral. Invest.* 18 1763–1769. 10.1007/s00784-013-1151-8 24297656

[B6] Ferrer-Espada R. Fang Y. Dai T. (2018). “Antimicrobial blue light inactivation of biofilms formed by clinical isolates of multidrug-resistant microorganisms,” in *Proc. SPIE 10479, Light-Based Diagnosis and Treatment of Infectious Diseases, 8 February 2018 2018*, (San Francisco).

[B7] Ferrer-Espada R. Wang Y. Go X. S. Dai T. (2020). Antimicrobial blue light inactivation of microbial isolates in biofilms. *Lasers Surg. Med.* 52 472–478. 10.1002/lsm.23159 31536154 PMC7080594

[B8] Gharaie S. Vaas L. A. I. Rosberg A. K. Windstam S. Karlsson M. E. Bergstrand K.-J. (2017). Light spectrum modifies the utilization pattern of energy sources in *Pseudomonas* sp. DR 5-09. *PLoS One* 12:e0189862. 10.1371/journal.pone.0189862 PMC5739431 29267321

[B9] Ghate V. S. Zhou W. Yuk H.-G. (2019). Perspectives and trends in the application of photodynamic inactivation for microbiological food safety. *Comprehens. Rev. Food Sci. Food Safety* 18 402–424. 10.1111/1541-4337.12418 33336937

[B10] Giannakis S. Rtimi S. Darakas E. Escalas-Cañellas A. Pulgarin C. (2015). Light wavelength-dependent E. coli survival changes after simulated solar disinfection of secondary effluent. *Photochem. Photobiolog. Sci.* 14:2238. 10.1039/c5pp00110b 26528694

[B11] Guffey J. S. Payne W. C. Motts S. D. Towery P. Hobson T. Harrell G. (2016). Inactivation of *Salmonella* on tainted foods: using blue light to disinfect cucumbers and processed meat products. *Food Sci. Nutrit.* 4 878–887. 10.1002/fsn3.354 27826438 PMC5090652

[B12] Higgins J. P. T. Savovic J. Page M. J. Elbers R. G. Sterne J. A. C. (2020). “Assessing risk of bias in a randomized trial,” in *Cochrane handbook for systematic reviews of interventions*, 2nd Edn, eds Higgins J. P. T. Thomas J. Chandler J. Cumpston M. Li T. Page M. J. (Chichester: John Wiley & Sons).

[B13] Higgins J. P. T. Thomas J. Chandler J. Cumpston M. Li T. Page M. J. (2019). *Cochrane handbook for systematic reviews of interventions.* Hoboken, NJ: Wiley Blackwell.

[B14] Hijnen W. A. M. Beerendonk E. F. Smedema G. J. (2006). Inactivation credit of UV radiation for viruses, bacteria and protozoan (oo)cysts in water: a review. *Water Res.* 40 3–22. 16386286 10.1016/j.watres.2005.10.030

[B15] Hoenes K. Stangl F. Stift M. Hessling M. (2015). “Visible optical radiation generates bactericidal effect applicable for inactivation of health care associated germs demonstrated by inactivation of *E. coli* and *B. subtilis* using 405 nm and 460 nm light emitting diodes,” in *Novel Biophotonics Techniques and Applications III*, eds Amelink A. Vitkin I. A. (SPIE-OSA), 1–9.

[B16] Hong C. Moorman G. W. Wohanka W. Büttner C. (2014). *Biology, detection, and management of plant pathogens in irrigation water*, St. Paul, MN: APS.

[B17] Hyun J.-E. Lee S.-Y. (2020). Blue light-emitting diodes as eco-friendly non-thermal technology in food preservation. *Trends Food Sci. Technol.* 105 284–295. 10.1016/j.tifs.2020.09.008

[B18] Hyun J.-E. Moon S.-K. Lee S.-Y. (2021). Antibacterial activity and mechanism of 460–470 nm light-emitting diodes against pathogenic bacteria and spoilage bacteria at different temperatures. *Food Control.* 123:107721. 10.1016/j.foodcont.2020.107721

[B19] Josewin S. W. Kim M.-J. Yuk H.-G. (2018). Inactivation of Listeria monocytogenes and *Salmonella* spp. on cantaloupe rinds by blue light emitting diodes (LEDs). *Food Microbiol.* 76 219–225. 10.1016/j.fm.2018.05.012 30166145

[B20] Kim D.-K. Kang D.-H. (2021). Efficacy of light-emitting diodes emitting 395, 405, 415, and 425 nm blue light for bacterial inactivation and the microbicidal mechanism. *Food Res. Internat.* 141:110105. 10.1016/j.foodres.2021.110105 33641972

[B21] Koutchma T. (2008). UV light for processing food. *Ozone: Sci. Eng.* 30 93–98. 10.1080/01919510701816346

[B22] Koutchma T. (2018). *Status of international regulations for ultraviolet treatment of foods. IUVA* 2018:Second Quarter. 13–16.

[B23] Krauss U. (2007). *Bacterial blue-light photoreceptors of the LOV family.* PhD. Düsseldorf: Heinrich Heine Univeristy.

[B24] Lacombe A. Niemira B. A. Sites J. Boyd G. Gurtler J. B. Tyrell B. (2016). Reduction of bacterial pathogens and potential surrogates on the surface of almonds using high-intensity 405-nanometer light. *J. Food Protect.* 79 1840–1845. 10.4315/0362-028X.JFP-15-418 28221904

[B25] Lewis K. (2010). Persister cells. *Annu. Rev. Microbiol.* 64 357–372. 10.1146/annurev.micro.112408.134306 20528688

[B26] Lui G. Y. Roser D. Corkish R. Ashbolt N. Stuetz R. (2016). Point-of-use water disinfection using ultraviolet and visible light-emitting diodes. *Sci. Total Env.* 553 626–635. 26967007 10.1016/j.scitotenv.2016.02.039

[B27] Maclean M. Macgregor S. J. Anderson J. G. Woolsey G. (2009). Inactivation of bacterial pathogens following exposure to light from a 405-nanometer light-emitting diode array. *Appl. Env. Microbiol.* 75 1932–1937. 10.1128/AEM.01892-08 19201962 PMC2663198

[B28] Marasini S. Zhang A. C. Dean S. J. Swift S. Craig J. P. (2021). Safety and efficacy of UV application for superficial infections in humans: a systematic review and meta-analysis. *Ocular Surface* 2021:002. 10.1016/j.jtos.2021.03.002 33812086

[B29] Moeyaert M. Ugille M. Beretvas S. N. Ferron J. Bunuan R. Van Den Noortgate W. (2017). Methods for dealing with multiple outcomes in meta-analysis: a comparison between averaging effect sizes, robust variance estimation and multilevel meta-analysis. *Internat. J. Soc. Res. Methodol.* 20:1252189. 10.1080/13645579.2016.1252189

[B30] Neal J. A. Marquez-Gonzalez M. Cabrera-Diaz E. Lucia L. M. O’bryan C. A. Crandall P. G. (2012). Comparison of multiple chemical sanitizers for reducing *Salmonella* and *Escherichia coli* O157:H7 on spinach (Spinacia oleracea) leaves. *Food Res. Internat.* 45 1123–1128. 10.1016/j.foodres.2011.04.011

[B31] Oliver J. D. (2010). Recent findings on the viable but nonculturable state in pathogenic bacteria. *FEMS Microbiol. Rev.* 34 415–425. 10.1111/j.1574-6976.2009.00200.x 20059548

[B32] Plavskii V. Y. Mikulich A. V. Tretyakova A. I Leusenka I. A. Plavskaya L. G. Kazyuchits O. A. (2018). Porphyrins and flavins as endogenous acceptors of optical radiation of blue spectral region determining photoinactivation of microbial cells. *J. Photochem. Photobiol. B: Biol.* 183 172–183. 10.1016/j.jphotobiol.2018.04.021 29715591

[B33] Pullerits K. Ahlinder J. Holmer L. Salononsson E. Öhrman C. Jacobsson K. (2020). Impact of UV irradiation at full scale on bacterial communities in drinking water. *npj Clean Water* 3:11. 10.1038/s41545-020-0057-7

[B34] Ryter S. W. Hong P. K. Hoetzl A. Park J. W. Nakahira K. Wang X. (2007). Mechanisms of cell death in oxidative stress. *Antioxid. Redox Signal.* 9 49–89. 10.1089/ars.2007.9.49 17115887

[B35] Song K. Mohseni M. Taghipour F. (2016). Application of ultraviolet light-emitting diodes (UV-LEDs) for water disinfection: a review. *Water Res.* 94 341–349. 26971809 10.1016/j.watres.2016.03.003

[B36] Tufanaru C. Munn Z. Stephenson M. Aromataris E. (2015). Fixed or random effects meta-analysis? Common methodological issues in systematic reviews of effectiveness. *Internat. J. Evid. Based Healthcare* 13 196–207. 10.1097/XEB.0000000000000065 26355603

[B37] Vermeulen N. Keeler W. J. Nandakumar K. Leung K. T. (2008). The bactericidal effect of ultraviolet and visible light on *Escherichia* col. *Biotechnol. Bioeng.* 99:21611. 10.1002/bit.21611 17680675

