# Supplementary material for: Blue Light Inhibits E. coli, but Decisive Parameters Remain Hidden in the Dark: Systematic Review and Meta-Analysis
Source: Front Microbiol. 2022 Apr 8;13:867865. doi: 10.3389/fmicb.2022.867865 (PMC9023763; doi:10.3389/fmicb.2022.867865)
Supplement: Supplementary file 1 [file Table_1.DOCX]

# Supplementary Table 1. Search query

("bluelight" OR "blue-light" OR "blue light") AND ("E. coli" OR "Escherichia coli")

Scopus

No time limitations

Number of articles: 430

Date of the data search: 15FEB21

All conversions need to be made

| Round 1 | | |
| --- | --- | --- |
| Accepted |  | 28 |
| Repeat |  | 1 |
| Catalyst |  | 75 |
| Cell division |  | 2 |
| Genetics |  | 12 |
| Heterologous Expression | | 28 |
| Motility |  | 8 |
| Not blue light or wavelength | | 7 |
| Not E. coli |  | 11 |
| Not inhibition | | 20 |
| Protein |  | 53 |
| title |  | 177 |
| Access/Conference/Language | | 8 |
|  |  |  |
| Total |  | 430 |
|  |  |  |
| Round 2 | | |
| Iinitial read through | | 28 |
| Removed |  | 7 |
| Missing SDs |  | 2 |
| Missing initial/final CFU/log or SDs or one or more | | 10 |
|  |  |  |
| Total |  | 9 |
