# Supplementary material for: Blue Light Inhibits E. coli, but Decisive Parameters Remain Hidden in the Dark: Systematic Review and Meta-Analysis
Source: Front Microbiol. 2022 Apr 8;13:867865. doi: 10.3389/fmicb.2022.867865 (PMC9023763; doi:10.3389/fmicb.2022.867865)
Supplement: Supplementary file 2 [file Table_2.DOCX]

Supplementary Table S2. Criteria underlying the translation of screening results to judgements (Higgins et al. 2020)

| **Low risk of bias** | **Some concerns** | **High risk of bias** |
| --- | --- | --- |
| the trial is judged to be of low risk of bias for all domains for this result | the trial is judged to raise some concerns in at least one domain for this result. But not to be at high risk of bias for any of the domains | the trial is judged to be at high risk for at least one domain for the result OR The trial is judged to have some concerns for multiple domains in a way that substantially lowers confidence in the result |
